# Medical Emergencies and Operational Preparedness Among Dentists: A Scoping Review

**DOI:** 10.3390/dj14040190

**Published:** 2026-03-24

**Authors:** Radu-Alexandru Iacobescu, Teofil Blaga, Raluca Dragomir, Ștefania-Crina Mihai, Petruța Moroșan, Anca Hăisan

**Affiliations:** 1 Grigore T. Popa University of Medicine and Pharmacy, 700115 Iasi, Romania; radu_iacobescu@yahoo.com (R.-A.I.); raluca.dragomir@umfiasi.ro (R.D.); stefaniacrina.mihai@gmail.com (Ș.-C.M.); petruta-anca.morosan@umfiasi.ro (P.M.); anca.haisan@umfiasi.ro (A.H.); 2 Emergency Department, “Sf. Spiridon” County Clinical Emergency Hospital, 700111 Iasi, Romania

**Keywords:** medical emergency, dentistry, emergency preparedness, emergency training, emergency frameworks, emergency epidemiology

## Abstract

**Background**: Medical emergencies occur at varying rates across the globe. Given the significant effort invested in identifying them and assessing dentists’ preparedness to deliver treatment in these life-threatening conditions, a global overview was needed. **Materials and Methods**: In this scoping review, data from PubMed, Cochrane Library, and Google Scholar databases were examined to identify all relevant studies reporting on the impact of medical emergencies on dentists and determine their operational preparedness at a national or regional level. Operational preparedness was determined in accordance with existing emergency operational preparedness frameworks across six domains: Anticipate, Assess, Prevent, Prepare, Respond, and Recover. **Significant Findings:** Global data show that dentists will invariably encounter medical emergencies across their careers. However, our investigation found that in countries where there is strong foundational training and regular refresher training, fewer frequent emergencies and stronger operational preparedness are reported. Governmental regulation emerged as a key facilitator of operational preparedness. Still, barriers exist, primarily limited access to medical emergency courses, shortages of office supplies for emergency drugs and materials, and the absence of medical emergency registries. **Conclusions**: A reassessment of the medical emergency training courses’ content appropriateness is paramount. Training interventions should also focus on raising awareness about the importance of preventive measures and office optimization through planning. Further research is needed to identify any overlooked facilitators and barriers to operational preparedness in medical emergencies. This will help identify opportunities for improvement and minimize the impact of emergencies on dental practices.

## 1. Introduction

Improvements in therapeutic procedures in dental care have increased the pool of patients suitable for complex treatments within acceptable safety margins; however, complications remain possible, and general urgent medical care may be required [1]. Although rare, medical emergencies are not uncommon, and a variety of medical emergencies impact the practice of dentistry specialists worldwide [2]. This is attributable to the wide range of patient ages, complex comorbidities, and the invasive nature of dental care, which together increase the risk of emergency care events [3].

Medical emergencies within dental practices are emerging as a significant global concern. Consequently, several international emergency societies have recommended incorporating training in first aid and emergency care for dentists [4,5]. For example, according to the ADA (American Dental Association), all dentists in the US are expected to safely manage medical emergencies and be prepared to act in various medical situations [5]. Still, university training varies with respect to content and methods, and coverage is not universal. In a previous scoping review by Vaughan et al., it was shown that, despite an overall positive attitude toward emergency training among dentistry students and practitioners, most studies reported a lack of skill and confidence in delivering emergency care following pregraduate training [6]. They also highlight that most training may not be adequately tailored to dental students’ professional needs and that there is a significant lag in implementing training practices. Further data about dentistry practitioners’ preparedness and exposure to emergency care have emerged but have not been properly assessed [7,8,9]. A global view of postgraduate dentists’ operational preparedness for emergency care is warranted.

There are no studies or frameworks that define medical emergency operational preparedness specifically for dentistry practitioners, but principles from existing emergency preparedness frameworks can be adapted [10,11]. Their operational domains usually include Anticipate, Assess, Prevent, Prepare, Respond, and Recover. A first step is to anticipate the common risks associated with the practices in their respective settings, followed by a risk assessment of the common events. Prevention is another cornerstone for reducing the impact of emergencies on dental practices [12]. This often includes accurate medical history-taking and questioning patients about prior emergency events [9]. A further step in developing operational preparedness is to ensure response plans within the practice and ensure access to emergency care materials and devices [3,13,14]. Another critical step is responding to emergencies, which require adequate knowledge and confidence to practice. Finally, recovery entails learning from past experiences and augmenting practice for future situations.

The goal of this scoping review is to summarize worldwide data on the frequency of emergency care among postgraduate dentistry practitioners and to document operational preparedness for emergency situations, with the aim of identifying additional improvement strategies.

## 2. Materials and Methods

This scoping review was conducted in accordance with the Preferred Reporting Items for Systematic Reviews and Meta-Analyses (PRISMA) from 2020 [15]. The PRISMA checklist for this review is available as Supplementary Table S1.

### 2.1. Identification of Relevant Studies

An extensive literature search was conducted using PubMed, the Cochrane Library, and Google Scholar to identify articles describing the frequency of emergency incidents among postgraduate dentistry practitioners and reporting operational preparedness components, up to 1 December 2025 (when the initial search was conducted). Further, the bibliographies of each study were screened to identify any missed literature. The search strategy combined Medical Subject Headings (MeSH) and free-text terms related to dental professionals, medical emergencies, and preparedness or competence. Boolean operators (AND/OR) were used to combine search concepts. The complete search strategy is provided in Supplementary Table S2.

### 2.2. Study Selection

Studies were included if they investigated cohorts at the national or at least regional level; consequently, single-center studies or studies reporting data from a single city were excluded. Only studies available as full text and in English or French were selected. Studies reporting on emergency care for pediatric patients were also excluded, as these differ significantly in training and practice standards for this age group and should be discussed separately. Data from cross-sectional studies reported in letters to the editor or posters were also not included in this review. No systematic assessment of research quality was conducted. This was done to enable the inclusion of as much epidemiologic data as possible to enrich the global perspective on emergencies in dental practice.

### 2.3. Data Extraction

The data were extracted by the two main investigators, R.-A.I. and T.B., into two categories: studies reporting on medical emergency prevalence and/or studies reporting on operational preparedness. Operational preparedness was assessed and reported according to the operational preparedness framework domains: Anticipate and Assess, Prevent, Prepare, Respond, and Recover. Extracted data contained sample characteristics, emergency frequency, exposure to training, use of preventive measures, availability of emergency drugs and materials, and self-reported confidence/competence measurements.

## 3. Results

**Figure 1:**
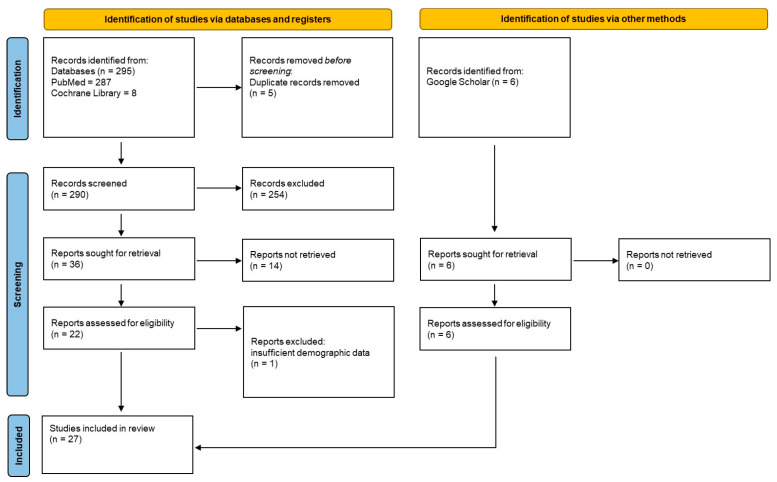
Study selection flow chart.

The initial PubMed and Cochrane Library search yielded 295 reports; after removing duplicates and irrelevant reports based on title and abstract, 36 articles were selected for full-text review. An additional 6 gray literature sources were identified using Google Scholar. Finally, 14 sources reporting on limited populations and not meeting the inclusion criteria were identified; thus, 27 studies were included in this review, reporting on 20 countries. A flow diagram of the study selection process is shown in Figure 1, and a summary of the study’s sample characteristics is available in Supplementary Table S3.

### 3.1. Anticipate—Epidemiology and Nature of Medical Emergencies in Dental Practices

**Figure 2:**
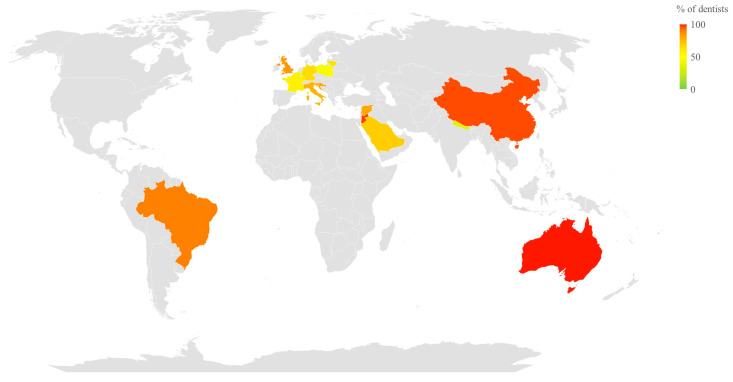
Prevalence of medical emergencies among dentists. The scale shows the percentage of survey respondents who reported having experienced a medical emergency. Some of the plotted data correspond to shorter time intervals.

An assessment of common medical emergency risks in dental care has been conducted nationally, or at least regionally, in only 19 countries. A summary of these findings is reported in Table 1. Most studies examined the frequency of emergency situations across participants’ entire careers (or reported as within the past 40 years of practice) [1,8,9,16,17,18,19,20,21,22,23,24,25]. The rates reported globally over the entirety of the career ranged between 37.1% and 94.3%, with an average of 71.1%, the lowest being reported in Belgium and Nepal (43.6% and 37.1%, respectively), and the highest in France, Australia, and Slovenia (94.3%, 94%, and 93.4%, respectively). Other studies have reported the proportion of dentists who encountered an emergency in the previous year [20,21,25,26,27,28,29], in the previous 10 years [29,30], or over some other interval [1]. This was done to reduce recall bias in the samples, and reporting for the previous year is likely more appropriate for accurately describing the density of emergencies among dentists. Reported rates for one year ranged from 53.2% (France) to 87.8% (Jordan), with a mean of 68.1%. The reported means are computed by averaging the data for each respective time interval. Figure 2 illustrates global event-density data by country, as reported in their respective studies, revealing several hotspots. Other articles reported on the overall incidence as a number of cases/dentist/year [7,20,24,25,26,31]. This proportion was reported to range from 0.38 (India) to 2.37 (Scotland-United Kingdom).

Some data reports participants’ experiences both within and outside the office. For instance, in Arterton’s study in the UK, 15.6% of the events happened outside the dental office, mostly allergic reactions, but 10.8% of the total cardiac arrests were also reported [24]. In Laurent et al.’s study of the French-speaking population in France and Belgium, 33.7% of participants stated cardiac arrest occurred in the office, 57.9% occurred outside the office, and 8.4% noted an event in both settings [20]. This proportion is also supported by Al Ghanam’s study in Jordan, which found 52.4% cardiac arrest events happened during office hours and 47.9% outside the work environment [21].

Regarding specific emergencies, syncope and presyncope are by far the most frequently reported events within dentists’ offices. Up to and exceeding half of the participants in almost all studies have identified this as the primary type of emergency in their practice. Other emergencies of matter in order of importance were diabetic-related (hypo/hyperglycemia), cardiovascular-related disease (Hypo/hypertension, Myocardial infarction), asthma-related events, and neurologic conditions (seizures and stroke). Severe life-threatening emergency situations are fortunately rare. However, throughout the professional career, the probability of a severe emergency remains elevated. Chapman et al. show that a non-negligible proportion of dentists will have to resuscitate patients during their lifetime, as one out of seven Australians has reported at least one such situation during their practice [16]. In Jordan, one in five participants has witnessed a cardiac arrest event, while almost half have had a major emergency event [21]. Death within the office has, however, been rarely reported, only 0.9% or less [1,18].

### 3.2. Assess—Medical Emergency Risk Assessment Practices and Clinical Screening

Studies reporting on operational preparedness have rarely considered the preinterventional assessment domain, and only nine studies have been identified, as reported in Table 2. Risk assessment in these studies includes preinterventional medical history evaluations, vital signs measurements, and clinical examinations, whereas no studies have been identified to report on structured risk assessment in dental practices.

Nine studies describe dentists’ practices on medical history-taking [17,19,28,33]. The best-performing samples in this regard were identified in more recent studies in countries such as India and Saudi Arabia, with proportions exceeding 90% [23,33,34,35]. In the remaining surveys, the proportion of participants taking a medical history was approximately 50%, and most reported taking it primarily orally.

Only six studies investigated preinterventional vital sign measuring or recording [8,23,28,33,34,35]. The proportion of dentists taking vital signs at any point within their practice ranged from 11.2% (Poland) to 42.4% (Saudi Arabia), with a mean of 30.7% across the included literature. The reported rates for measuring every time were even lower, ranging from 0.96% (Poland) to 11% (Saudi Arabia), with an average of 5.9%. Among the survey participants, no difference was observed between specialities [35]. However, this practice was more common in the hospital setting. According to Kaddah’s study, 30.7% of the whole sample reporting taking vitals were from hospitals, compared to 28.3% in independent offices (*p* < 0.001) [8].

Only one study investigates clinical examination practices, mainly Jabers et al.’s study, which found that medical clinical examination is the norm before any procedure for 89% of survey participants [23].

### 3.3. Prevent—Strategies to Reduce Emergency Occurrence

Prevention within the dental medical emergency framework refers to whether case selection is based on history and whether treatment is modified based on the risk assessment. Although several studies have reported on the practice of medical history taking, it is often unclear whether the information obtained was used to select patients for further programmed intervention or whether the intervention was postponed until the underlying conditions were optimized. Only one study addressed this issue. In Saudi Arabia, the most recent published national cross-sectional survey found that 94% of participants reported adjusting treatment based on the clinical examination and history provided [23].

### 3.4. Prepare—Structural and Organizational Readiness

There are several investigations regarding dental practices’ preparedness, summarized in Table 3. These refer to the availability of emergency kits, emergency drugs, emergency devices, and emergency plans. Provisions for medical emergency supplies have been described as being regulated in only 4 countries in their respective studies (Germany [27], New Zealand [29], Saudi Arabia [32], and the United Kingdom [36]). Not surprisingly, a proper emergency kit is reported in up to a third of respondents in countries with less stringent emergency preparedness regulations (India [33], Lebanon [9], Jordan [21], Lithuania [30], and Nepal [22]) versus approximately 80% in countries where such regulations exist.

The types of emergency supplies and drugs have been variously reported. The most frequently identified emergency medical devices were blood pressure monitors, glucometers, oxygen supply and mask, airway management devices, and AEDs. Oxygen supply was reported in 13 studies, with provisions found to be as low as 14.6% of survey participants (Syria [8]) and up to 94% (Jordan [21]), with a mean of 51.2%. Results from 9 investigations show that blood pressure measuring devices are available in up to half of dental offices, ranging from 2% (Australia [16]) and up to 54.8% (India [7]) with a mean of 34%. The existence of glucometers within offices was investigated in only three studies, with reports ranging from 25.7% (Saudi Arabia [34]) up to 41.2% (India [7]) averaging at 31.1%. Regarding airway management devices, several have been considered, ranging from pocket masks to supraglottic devices, and have been summarized in Table 3. Lastly, the existence of AEDs has been assessed in 10 surveys, with provisions ranging from 0% (Syria [8]) to 26% (Saudi Arabia [23]) with an average of 9.43%. This number remains critically low, and, except in Poland and Slovenia, the provision of this life-saving device was reported in fewer than 10% of offices [26,28]. An increase in this matter across studies in Saudi Arabia, ranging from 10% to 26%, suggests efforts toward office appropriation [23,34].

Emergency drugs have been investigated in 14 studies. The type of emergency drug varies even across studies that document the same population [23,34]. The most commonly identified are adrenaline, corticosteroids, and antihypoglycemic medications. Adrenalin was the most abundant emergency drug in 10 of the included surveys, ranging from 22% (Australia [16]) to 98.7% (Croatia [37]) averaging 66%. This is most likely because of the utility it plays in daily practice. Corticosteroids were identified in seven surveys, ranging from 9% (Australia [16]) to 87.8% (Croatia [37]) with an average of 48.7%. Oral glucose was reported by 8 studies, ranging from 6.9% (Croatia [37]) to 81.4% (India [33]) with an average of 53.1%, while intravenous glucose solutions were available only to 4% and up to 22.2% according to two reports [16,29], and glucagon (a critical rescue medication in severe hypoglycemia) was available to only 9.4% and 30%, as reported by only two studies [34,37].

Emergency care protocols are rarely reported within practices by surveys. Data were available in only seven studies from six countries [7,8,9,21,23,29,34]. The existence of plans among dentists’ offices ranges from 8.8% (Syria [8]) to 68% (Saudi Arabia [23]). Again, in the case of Saudi Arabia, a noticeable increase is evident across older and more recent studies, suggesting an improvement in practice standards from 20% to 68% [23,34].

### 3.5. Respond—Competence and Confidence in Managing Medical Emergencies

Medical emergency self-reported competence or confidence to respond was investigated by 25 studies. This included confidence in diagnosing medical emergencies (evaluated in five studies), confidence or competence in treating or intervening in emergency situations (reported in 20 surveys), and confidence in performing life-saving procedures (assessed in 14 studies). A summary of these findings is available in Supplementary Table S4.

Studies indicate a significant disconnect between dentists’ self-confidence in diagnosing medical emergencies and their self-perceived competence [26]. Generally, lower confidence in diagnosing medical emergencies is reported [25,26]. Notable exceptions are Čuković-Bagić’s study from Croatia, where 79.5% of participants said they can diagnose emergency conditions, while up to half reported being able to treat one, and Jaber’s study from Saudi Arabia, where more participants were confident in diagnosing an emergency (50%) than managing one (48%) [19,23].

When assessing confidence and competence in managing medical emergencies, most studies reported low levels, with notable exceptions among studies on practitioners from France, India, Syria, and the United Kingdom. In a survey on preparedness for emergencies in India, Gupta et al. found that 77.4% of respondents declared competence in performing CPR, while 69.9% said they were competent in handling emergencies [38]. This is paradoxical, as in their sample, 62.3% of respondents stated never having received BLS training. In a previous analysis of preparedness for medical emergencies in two regions of the country, confidence and training were similar: 94% of respondents declared reasonable confidence in resolving an emergency situation, whereas only 7.6% were trained to deliver any type of emergency care [33]. Sampling a single center in India, Fernandez A.L.V.C. et al. found that 46.6% expressed confidence in emergency management, with 17.86% of the surveyed population having been trained [39]. They also point out that most of the sample was uninterested in emergency care and had only moderate knowledge. Overestimation of one’s self-confidence is thus highly probable, and data on confidence or competence should be interpreted cautiously. Confidence in treating emergencies is also reported to vary by the type of emergency considered. Expectably, survey participants felt more confident in treating minor emergencies such as faints, hyperventilation crises, or hypoglycemia rather than severe ones like angina, asthma attacks, anaphylaxis, stroke, convulsions, or myocardial infarction [1,9,17,19,20,21,25,26,28,37].

Some studies investigated competence or confidence in performing CPR specifically. Percentages of survey responders declaring competence were less variable in this regard: 77.4% (India [38]), 55% (Australia [16]), 50.6% (Slovenia [26]), 49% (Germany [27]), 47.3% (India [7]), 44.8% (Saudi Arabia [32]), with the sampled data averaging 54%. Other procedures, such as advanced life support, airway management, or other ventilation maneuvers and defibrillation, have been rarely reported (only by three studies) [7,27,31]. They show that the self-reported proficiency in performing these maneuvers is usually very low. For instance, in Müller et al.’s study, self-reported competence in performing airway management was 16%, while confidence in defibrillation was reported only by 3% of responders [27]. Confidence in administering medication was presented in nine studies. These studies show variable results, with less than half feeling confident in administering medication in five studies [22,25,26,30,33], and more than half expressing elevated confidence in four studies [7,29,35,37]. Two studies note, however, that there is a significant difference in confidence displayed depending on the route of administration, highlighting that dentists are more comfortable with intramuscular than intravenous administration [33,35]. Lastly, confidence in using medical devices was explored by two studies, with less than half of the responders feeling confident [30,37].

### 3.6. Recover—Post-Event Learning and Practice Improvement

Recovery is primarily focused on learning from past experiences, encompassing documentation and continuous improvement efforts. To draw lessons from past experiences, some form of record must be kept. Dentists are mandated to record patients’ visits. However, a single study reported on medical emergency records within dentists’ offices. In the most recent study included in this review from Syria, 67.5% of participants declared accurate documentation of emergency situations that occurred within the workplace [8]. In Varoni et al.’s study in Italy responders showed support for a national registry of medical emergencies in dentistry [1].

There is significant skill decay in emergency management, and refresher courses are required for all specialists annually [40,41]. Not surprisingly, Müller et al.’s study shows no difference in confidence in emergency management between survey participants with no training and those who participated in only one session [27]. However, greater confidence was reported among participants who had multiple instruction sessions, underlining the importance of repeated instruction for improving confidence. Reports from Belgium and Germany also support these findings. In these studies, those who had more than one training session reported better competence in diagnosing and treating medical emergencies than those with only one session or no training [17,27].

## 4. Discussion

Our investigation shows that the burden of medical emergencies in dental practices is significant, and the likelihood of experiencing an emergency at some point in a career—if not every year—is virtually certain. There are noticeable differences between countries in this area, likely due to prevention practices and training. We argue that some observed differences between countries are attributable to differences in investigation methodology, because the populations described by profession, place of practice, and workplace location vary significantly across studies, as reported in Supplementary Table S3. Emergencies are more likely within hospital settings and with more invasive procedures such as surgery. Furthermore, the reported proportions are influenced by the number of patients a physician assesses in a given year or over their career, and this manner of reporting prevalence may be biased. Studies reporting medical emergencies per physician per year provide a more accurate perspective on the medical emergency burden, but are scarcer. Furthermore, some studies also reported on out-of-office medical emergencies, while others might not have accounted for this distinction. Given this, some reported results might be overstated for medical emergencies within the office. However, this goes to show the importance of emergency care training beyond the confines of the job and its applicability to daily life, as emergencies affect people ubiquitously. Another reason for the difference in prevalence stems from the medical emergencies considered. Some investigations have deliberately asked participants whether any emergency occurred within the given timeframe, while others have simply investigated specific emergencies. Since the studies accounted for between 4 and 17 medical emergencies, the reported frequencies may be underreported in some instances [19,22,27,30]. We note that the studies included in our analysis span a substantial time period; consequently, practices and safety may have improved since publication, and their findings may no longer be relevant.

The data presented show that some medical emergencies occur more often than others. This means that all dentists should have a firm grasp on the etiology and treatment of these medical emergencies, such as syncope [42]. Consequently, relevant training programs should focus specifically on addressing them. Fortunately, most of these are not serious conditions and rarely pose significant risks to life. Still, the reported proportion of dentists involved in at least one instance of cardiac resuscitation is not negligible. This review’s findings indicate a lack of confidence and competence in diagnosing and treating emergency conditions among a large proportion of dentists, particularly for more serious conditions. Consequently, we argue that the curricular content for emergency medical training may not be sufficiently focused and should specifically target the identified vulnerabilities.

Training in the provision of care for these life-threatening emergencies is pivotal and can significantly impact outcomes. Training practices vary across countries in terms of content, duration, and instructional methods. Despite the great support of respondents for hands-on courses rather than lecture-based teaching, studies reported limited use of roleplay, mannequin practice, or other hands-on experiences [7,38]. Lecture-based teaching offers several advantages, such as accessibility to large audiences and low costs; however, it may fall short in conveying knowledge, competence, and confidence. Another difference is pre-graduate access to training. In countries like Germany, Poland, Slovenia, Saudi Arabia, and the UK, pregraduate training was mandatory at the time of reporting. Proportions of pregraduate training observed in studies without mandatory training in dentistry universities are attributable to teachers’ dedication to providing education through workshops and supplementary courses. Postgraduate training is also mandatory in a few instances; however, it largely depends on the individual’s interest. Notably, BLS skills tend to decline quickly in this professional group due to infrequent use in practice; therefore, refresher courses at least every two years (and generally annually) have been recommended to maintain proficiency [40,43]. A recent educational intervention study based on frequent emergency scenarios encountered in the dental office, such as syncope, anaphylaxis, hyperventilation, and acute coronary syndrome, found that skill decay occurred even more rapidly, and participants lost significant skills even after three months [44]. The optimal frequency of refresher courses remains debatable; however, regular assessment of skills in emergency management should be undertaken to ensure minimal practice standards and to optimize practice in line with the latest standards [45].

Some estimates indicate that up to 90% of medical emergencies could be avoided through accurate selection of cases, informed by detailed history-taking, accurate measurement of vital signs, and appropriate planning of dental procedures [39,46]. Despite the importance of patient history utilization in reducing the emergency care burden, studies have rarely reported on its use. Saudi Arabia stands out in this regard, as the low reported incidence is most likely due to stringent governmental regulations governing dental practices and high uptake of preventive strategies. In Jaber’s study, 94% of respondents reported that patients’ medical history information was appropriately used to adjust treatment plans [23]. There is a longstanding tradition of emergency training for dentists in this country, and this has yielded better outcomes than in neighboring countries [47]. However, even in the aforementioned study, fewer than half of the participants reported confidence in their ability to prevent a medical emergency [23]. Our analysis also showed a positive trend in recent publications on medical history-taking. This may suggest greater uptake of preventive practices in recent years and improved awareness. Some studies also show that younger generations are more likely to obtain a medical history before procedures. In a small-sample research from the United Arab Emirates comprising predominantly general dentistry practitioners with less than 10 years of experience, 95% of participants reported obtaining a formal medical patient history [35]. This suggests a shift in global dental care practice, which is commendable. Uptake of this preventive practice might be limited because obtaining an accurate medical history is often challenging in the time-limited interactions between dentists and patients. Thus, tools are required to facilitate this task even for the less experienced doctors. A standardized risk assessment questionnaire has previously been developed for this context, and the validation study demonstrates good predictive performance for adverse outcomes in dental procedures [48]. This might represent a solution for the time-constrained context of private practice and for those inexperienced in taking medical histories. Despite its existence for several years, the uptake of this kind of tool has been scarce.

Taking vital signs has been strongly supported by the ADA and is a crucial component of the risk assessment pre-interventional process [49]. Vital signs, including blood pressure, pulse, respiratory rate, and body temperature, should be obtained before every procedure [50]. This has the potential to significantly reduce cardiovascular-related emergency risks, which, in our study, emerged as prevalent emergency conditions. Yet in the studies included in this review, the practice of measuring vital signs is even less frequently described. Taken together, the data indicate a strong need to adjust dental preventive practices. With a few notable exceptions, studies consistently reported limited uptake of medical history and vital signs assessment. These exceptions hint that training in emergency situations is an important opportunity to raise awareness among specialists about emergency prevention and has the potential to change practice, as countries with mandatory emergency training among dentistry specialists display higher proportions.

Our operational preparedness investigation also shows vulnerabilities in the office appropriation of emergency supplies and predefined emergency care planning in most instances. The recommended contents of an emergency kit are of limited relevance to the everyday practice of dentists [51]. Maintaining such a kit is a financial burden for most dentists and is often cited as a deterrent. The lack of various emergency equipment and drugs has been identified as a significant vulnerability in the workspace. Critically, access to an AED is most prominent. Despite international guidelines emphasizing the importance of AEDs in resuscitation, a substantial proportion of the evaluated studies reported limited access when needed. Consequently, a reassessment of the minimum standard emergency kit for dental offices is warranted, and we recommend that all regulatory institutions enforce adequate provisions for emergency supplies in all dental offices. Also concerning is that, regardless of their existence within the practice, dentists generally displayed a lack of confidence and knowledge in using emergency devices and medication [7,22,26,30,33,37]. Given this, training should not only address resuscitation but also encompass the use of devices and drugs and provide instruction on other medical procedures, such as airway management and intravenous drug administration. Furthermore, in-office medical emergency drills are recommended to optimize uptake and refine care according to each office setting. Having an already prepared plan for emergency situations can significantly lessen the impact these situations have on the dentistry practice and is also ubiquitously recommended [3,13].

Studies included in this review are limited by their design. Several studies might not be representative of the entire population due to the sampling strategy used, such as those that sampled participants at national conferences or other events [17,22,25]. In studies that investigated the entire dentist population of the region or country, the response rates for the survey are low, and thus, the results might also misrepresent the population [1,9,20,26,27]. Several studies did not describe the sampling strategy, which undermines credibility in sample representativeness [19,21,24,28,31,35,36,38,41]. Furthermore, most surveys use different unstandardized questionnaires, and items vary from one study to another; consequently, comparisons between studies remain limited, and data should be interpreted cautiously. Data is based on participants’ recollection of events for a given time period. To obtain accurate information, analyses of register data are necessary, which have not been performed at the national or regional scale. Reports on participants’ confidence and competence are also subject to bias. Specifically, self-reported measurements are influenced by social desirability, the Dunning-Kruger effect [52]. In studies included in this review, determination of confidence has been reported differently, measured using scores, or assessed using Likert scale questions, and the results of these studies might not be comparable. A tool to assess this domain would be highly beneficial. One such instrument was developed by Alawaji Y.N. et al. for more accurate detection of dentists’ confidence and self-readiness, which has been validated and is thus recommended for future studies [53].

## 5. Conclusions

Globally, medical emergencies affect a substantial proportion of dentists annually, and most will encounter one during their careers. Overall operational preparedness for medical emergencies among dentists is generally modest, as globally more than half of dentists report limited use of risk assessment, lack of preventive practices and emergency provisions, and low confidence in their capacity to respond to an emergency situation. Adequate and repeated training in undergraduate and postgraduate settings is a major influencer of operational preparedness. Problems remain in ensuring the adequacy and relevance of training, as well as its coverage, which could be addressed through appropriate legislative support.

## Figures and Tables

**Table 1:** Medical emergency prevalence in dentistry practice.

Author [Ref.], Year, Country.	Overall Prevalence	Specific Emergencies Prevalence
Choufani et al. [9], 2025, Lebanon.	74.2% encountered at least one minor medical emergency; 22.4% encountered a major medical event;	1. Vasovagal Syndrome 56.5% 2. Hypoglycemia 55.9% 3. Orthostatic hypotension 54.0% 4. Allergic reactions 13.0% 5. Hyperventilation crisis 11.2% 6. Hypertensive crisis 9.3% 7. Convulsions 5.6% 8. Asthma attack 4.3% 9. Ingestion of foreign bodies 4.3% 10. Inhalation of foreign body 3.51% 11. Severe allergic reaction 1.9% 12. Cardiac arrest 1.9% 13. Stroke angina/myocardial infarction 1.2%
Subhadra H.N. et al. [7], 2025, India.	0.38 emergencies/dentist/year;	1. Vasovagal Syncope 45.6% 2. Aspiration/ingestion of foreign body 15.2% 3. Hypoglycemia 12.1% 4. Mild allergic reactions 10.5% 5. Seizure 10.4% 6. Asthma attack 2.6% 7. Local anesthetic overdose 1.3% 8. Hyperventilation 1% 9. Anaphylaxis 0.7% 10. Cardiac arrest 0.5%
Kaddah M. et al. [8], 2025, Syria.	67.5% encountered at least a complication;	1. Vasovagal syncope 48.6% 2. Hypoglycemia 42.9% 3. Seizures 31.4% 4. Asthma attack 17.1% 5. Hypertension 20% 6. Severe allergic reactions 14.3% 7. Bleeding 9.8%
Varoni et al. [1], 2023, Italy.	65.2% encountered at least one medical emergency during their practice; 21.3% at least one in the past two years; 0.9% reported that a patient died due to a medical emergency;	1. Vasovagal Syndrome 2. Hypoglycemic Crisis 3. Seizure 4. Hypertensive Crisis 5. Allergic Drug Reaction 6. Severe Bleeding 7. Foreign Body Ingestion
Sin M. et al. [31], 2023, United Kingdom.	NA	1. Syncope 0.6272/year 2. Non-specific collapse 0.6092/year 3. Hypoglycemia 0.1210/year 4. Seizure 0.0951/year 5. Hyperventilation 0.0576/year 6. Asthma 0.0490/year 7. Acute coronary syndrome 8. 0.0317/year 9. Angina 0.0288/year 10. Anaphylaxis 0.0086/year 11. Choking 0.0058/year 12. Adrenal crisis 0.0029/year 13. Cardiac arrest 0.0029
Al Ghanam et al. [21], 2022, Jordan.	87.8% at least one minor emergency within the previous 12 months; 43.9% reported at least one major emergency during their practice; 23.3% witnessed a cardiac arrest;	1. Vasovagal Syncope 45% 2. Hypoglycemia 37.2% 3. Orthostatic Hypotension 26.7% 4. Minor allergic reactions 14.4% 5. Mild/severe allergic reactions 19.4% 6. Convulsions 9.4% 7. Asthma attack 8.3%
Jaber L. et al. [23], 2021, Saudi Arabia.	60% experienced a medical emergency;	1. Vasovagal syncope 54% 2. Hypoglycaemia 45% 3. Seizure 14% 4. Bronchospasm 12% 5. Angina/myocardial infarction 7% 6. Anaphylaxis 6% 7. Respiratory arrest 4% 8. Stroke 3% 9. Cardiac arrest 2% 10. Thyrotoxic crisis 1%
Jing Q. [18] et al., 2020, China.	85% reported an emergency situation; 35.5% reported more than one;	1. Vasovagal Syncope 35.9% 2. Hypoglycemia 30.3% 3. Adrenalin reaction 8.55 4. Allergy 5.8% 5. Anesthesia reaction 4.2% 6. Foreign body aspiration/ingestion 4% 7. Hypertension 3.7% 8. Seizure 3.1% 9. Orthostatic Hypotension 3.1% 10. Angina/Myocardial infraction 0.7% 11. Airway obstruction 0.4% 12. Stroke 0.3% 13. Cardiac arrest and death 0.2%
Smereka et al. [28], 2019, Poland.	19.56% requested EMS aid at least once in the past year.	1. Vasovagal Syncope 46.3% 2. Orthostatic hypotension 18.85% 3. Hyperventilation Crisis 18.61% 4. Mild allergic reaction 16.23% 5. Hypoglycemia 15.99% 6. Seizures 11.21% 7. Angina 6.21% 8. Hypertensive Crisis 2.15% 9. Anaphylactic Shock 2.14% 10. Asthma 3.1% 11. Cardiac arrest 1.9%
Umek N. and Šoštarič M. [26], 2018, Slovenia.	67.5% at least one minor emergency within the previous 12 months; 39.8% reported 3 emergencies within the previous 12 months; 26.3% reported up to 10 emergencies within a year; 93.4% reported one across the entirety of their career;	1. Vasovagal Syncope 61.9% 2. Hypoglycemia 9.7% 3. Anaphylaxis 3.1% 4. Hyperventilation crisis 2.8% 5. Seizure 2.1% 6. Airway obstruction 1.4% 7. Asthma 1% 8. Acute coronary syndrome 0.7% 9. Stroke 0.4% 10. Cardiac arrest 0% 11. Other emergencies 6.2%
Čuković-Bagić I. et al. [19], 2017, Croatia.	68.7% experienced an emergency during their practice;	1. Vasovagal syncope 57.4% 2. Hypo/Hyperglycemia 25.7% 3. Seizures 23.5% 4. Anaphylaxis 17.3% 5. Cardiac arrest 5.6%
Geguzis et al. [30], 2016, Lithuania.	55.8% reported an emergency within the past 10 years	1. Vasovagal Syncope 46.6% 2. Orthostatic Hypotension 20.4% 3. Convulsions 16.7% 4. Hypoglycemia 8.7%
Müller et al. [27], 2015, Germany.	57% reported up to 3 emergencies per year; 36% reported up to 10 emergencies per year;	1. Vasovagal Syncope 57.7% 2. Seizure 6.8% 3. Hypertensive crisis 6.6% 4. Asthma 3.9% 5. Hypoglycemia 3.5% 6. Acute coronary syndrome 3.5%
Joshi S. et al. [22], 2015, Nepal.	37.1% encountered at least an emergency situation;	1. Syncope 71.7% 2. Hypoglycemia 6.5% 3. Seizure 4.4% 4. Allergic reaction 4.4%
Alhamad et al. [32], 2015, Saudi Arabia.	67% encountered at least an emergency situation across their career;	1. Vasovagal syncope 53.1% 2. Hypoglycemia 44.8% 3. Orthostatic hypotension 21.3% 4. Seizures 16.6% 5. Asthmatic attack 11% 6. Adverse drug reactions 7.8% 7. Heart-related problems 8.3% 8. Foreign body aspiration 5.5% 9. Other 7%
Laurent et al. [20], 2014, France.	53.2% at least one emergency within the previous 12 months; 94.3% reported at least one emergency during their career; 2.1 emergencies/dentist/year;	1. Vasovagal syncope 46.8% 2. Orthostatic hypotension 21% 3. Hypoglycemia 19.6% 4. Minor allergic reactions 8.8% 5. Hyperventilation crisis 9.4% 6. Ingestion of foreign bodies 4.1% 7. Asthma attack 2% 8. Hypertensive crisis 1.3% 9. Convulsions 1.5% 10. Serious allergic reaction 0.9% 11. Inhalation of foreign bodies 1% 12. Cardiac arrest 0.4% 13. Angina/Myocardial infarction 0.4% 14. Stroke 0.3%
Marks L. A. M. et al. [17], 2013, Belgium.	43.6% reported an emergency within the office;	1. Vasovagal syncope 34.3% 2. Hypo/hyperglycemia 8.4% 3. Seizure 16.1% 4. Anaphylaxis 3.8% 5. Cardiac arrest 0.4% 6. Undiagnosed emergency 9.3% 7. Others 6.2%
Arsati et al. [25], 2010, Brazil.	75% reported an emergency within the previous 12 months;	1. Vasovagal Syncope and presyncope 66.9% 2. Orthostatic Hypotension 44.4% 3. Moderate allergic reactions 16.86% 4. Hypertension crisis 15.06% 5. Asthma 15.06% 6. Angina 6.82% 7. Convulsion 6.22% 8. Hypoglycemia 5.62% 9. Hyperventilation crisis 5.22%
Broadbent J.M. and Thomson E.M. [29], 2001, New Zealand.	NA	Reported within the previous year 1. Vasovagal syncope 61.1% 2. Hyperventilation crises 27.8% Reported within the previous 10 years 3. Angina 14.6% 4. Hypotension 11.1% 5. Myocardial infarction 2.5% 6. Stroke 2.5% 7. Respiratory depression 17.25% 8. Choking 1.5% 9. Asthma attack 7.6% 10. Seizures 26.2% 11. Allergic reaction 30.3% 12. Anaphylaxis 3.5% 13. Hypoglycemia 20.7% 14. Swallowed/inhaled foreign body 20.7% 15. Anesthetic overdose 5.1% 16. Drug interaction 5.6% 17. Other 9.1%
Atherton G.J. et al. [24], 1999, United Kingdom.	NA	1. Syncope 0.6272/year 2. Non-specific collapse 0.6092/year 3. Hypoglycemia 0.1210/year 4. Seizure 0.0951/year 5. Hyperventilation 0.0576/year 6. Asthma 0.0490/year 7. Acute coronary syndrome 8. 0.0317/year 9. Angina 0.0288/year 10. Anaphylaxis 0.0086/year 11. Choking 0.0058/year 12. Adrenal crisis 0.0029/year 13. Cardiac arrest 0.0029
Chapman P.J. et al. [16], 1997, Australia.	94% mentioned an emergency across their entire career;	1. Local anesthetic reaction 39% 2. Seizures 13.9% 3. Angina 9.2% 4. Hypoglycemia 5.8% 5. Asthma 3.2% 6. Resuscitation 1.3% 7. Myocardial infarction 0.7% 8. Stroke 0.4% 9. Anaphylaxis 0.1%

NA = Data NOT AVAILABLE.

**Table 2:** Preinterventional risk assessment and clinical screening.

Author [Ref.]	Medical History	Vital Signs and Clinical Assessment
Marks L. A. M. et al. [17]	55.3% recorded medical history; 26.6% took medical history frequently; 27.7% updated medical history; 8.6% never took medical history;	NA
Čuković-Bagić I. et al. [19]	51.2% reported always taking medical history; 36.7% update history each visit; 10.4% never take history;	NA
Kumarswami S. et al. [33]	98% took medical history; 12% Written medical History;	38.4% recorded vital signs;
Smereka et al. [28]	Took medical History: 44.9% orally; 31% in writing; 24.1% either in writing or orally;	11.2% recorded vital signs; 0.96% always recorded vitals; 10.2% were likely to record vitals; 89% were unlikely to record or never recorded vitals;
Jaber L. et al. [23]	94% always obtained medical history;	19% reported taking vital signs; 90% performed visual inspection; 89% carried out medical inspection;
Al-Sebaei M.O. [34]	92% obtained medical history;	42.4% measured vital signs; 11% measured vital signs every visit; 34.4% measured vital signs at the initial visit; 57% never measured vital signs;
Umek N. and Šoštarič M. [26]	26% reported always taking medical history; 29.2% reported taking medical history only at the first visit;	NA
Kaddah M. et al. [8]	17.7% take written medical history; 50.1% take medical history occasionally; 32.2% never take medical history;	41% took vital signs; 5.8% always measured vital signs; 35.2% occasionally measured vitals; 32.6% never measured vitals;
Shaath H. et al. [35]	95% obtained patient history at the initial visit;	32% measured vital signs;

NA = Data NOT AVAILABLE.

**Table 3:** Structural and organizational readiness.

Author [Ref.], Year.	Complete Emergency Kit	Emergency Devices	Emergency Drugs	Emergency Plans
Chapman P.J. et al. [16], 1997.	First aid kit 3%	1. Oxygen supply 63% 2. Manual resuscitator 27% 3. Pocket mask 4% 4. Blood pressure monitor 2%	1. Adrenaline 22% 2. Bronchodilator spray 13% 3. Oral glucose 11% 4. Glyceryl trinitrate 5.tablets/spray 9% 6. Hydrocortisone injection 9% 7. Antihistamine injection 9% 8. Diazepam injection 5% 9. Atropine injection 5% 10. Glucose injection 4% 11. Aromatic ammonia 1%	NA
Čuković-Bagić I. et al. [19], 2017.	NA	1. Ambu-bag 47.4% 2. Oxygen mask 43.6% 3. Oxygen supply 33.7% 4. AED 6.4%	1. Adrenaline 92.6%	NA
Špiljak B. et al. [37], 2024.	NA	1. Ambu-bag 34.2% 2. Oxygen supply 29.1% 3. Breathing mask 27.3% 4. Oropharyngeal airway 14.4% 5. Nasal canula 8.5% 6. Laryngeal mask 3.8%	1. Adrenaline 98.7% 2. Antihistamine 96.9% 3. Corticosteroid 87.8% 4. Aminophylline 77.1% 5. Diazepam 51.1% 6. Aspirin 37% 7. Bronchodilator 32.6% 8. Nitroglycerine 24.4% 9. Glucagon 9.4% 10. Oral Glucose 6.9%	NA
Laurent et al. [20], 2014.	NA	1. Oxygen supply 66.8% 2. Bag mask 59.6% 3. Blood pressure measurement device 51% 4. Defibrillator 8.6%	Any emergency drugs 70.5%	NA
Müller et al. [27], 2015.	Emergency kit 84%	1. ventilation bag 88% 2. airway device 73% 3. oxygen supply 72% 4. defibrillator 2%	NA	NA
Subhadra H.N. et al. [7], 2025.	NA	1. Blood pressure device 54.8% 2. Glucose monitor 41.2% 3. I.V. line 30.4% 4. Portable suction 29.9% 5. Pocket mask 27.4% 6. Oxygen supply 23.6% 7. Oropharyngeal airway 21.7% 8. Self-inflating bag 21.7%	1. Oral glucose 79.5% 2. Adrenaline 50.4% 3. Glyceryl trinitrate 33.8% 4. Ammonia 35.1% 5. Bronchodilator 34.6% 6. Atropine 32.8% 7. Hydrocortisone 31.4% 8. Diazepam 28%	43.8% had written medical emergency protocols.
Kumarswami S. et al. [33], 2017.	Any emergency Kit 24%	NA	1. Adrenaline 90% 2. Oral glucose 81.4% 3. Ammonia inhalant 78.3% 4. Hydrocortisone 55% 5. Atropine 53.33% 6. Glyceryl trinitrate 11% 7. Bronchodilator spray 7%	NA
Al Ghanam et al. [21], 2022.	38.2% reported having an emergency kit available	37.8% had any emergency supply of which: 1. Oxygen supply 94% 2. Blood pressure monitor 18.3% 3. Defibrillator 4.4%	Any emergency medication 27.8%	36.1% had an emergency plan.
Choufani et al. [9], 2025.	NA	NA	NA	16.1% reported having an emergency response plan;
Geguzis et al. [30], 2016.	36.2% had an emergency kit	1. Blood pressure monitor 16.5%	1. Adrenaline 28% 2. Prednisolone 9.2%	NA
Joshi S. et al. [22], 2015.	NA	46.8% have medical equipment	46.8% have emergency medication	NA
Broadbent J.M. and Thomson E.M. [29], 2001.	80.8% had an emergency kit	1. Suction 93.9% 2. Ambubag 81.8% 3. Oxygen apparatus 75.3% 4. Tourniquet 54.5% 5. Oxygen-nitrous oxide apparatus 53% 6. Paper bag 52.5% 7. Blood-pressure monitor 50.5% 8. Intravenous cannula 46.5% 9. Pulse oximeter 41.4% 10. Apparatus for delivering air 34.8% 11. Laryngoscope 24.7% 12. Other equipment 5.1% 13. ECG monitor 4.0% 14. Defibrillator 3.5%	1. Oxygen 78.8% 2. Adrenaline 68.2% 3. Oral glucose 60.6% 4. Nitrous oxide 55.6% 5. Salbutamol 55.1% 6. Flumazenil 36.4% 7. Midazolam 35.4% 8. Other benzodiazepine 22.2% 9. IV glucose 22.2% 10. Naloxone 17.2% 11. Other drugs 11.6%	63.1% had a written emergency plan;
Smereka et al. [28], 2019.	NA	1. Self-inflatable bag 82.3% 2. Oropharyngeal Airway 64.91% 3. Pocket mask 39.1% 4. Supraglottic Airway devices 35.8% 5. Portable suction device 22.9% 6. Oxygen supply 21.48% 7. AED 17.9%	NA	NA
Jaber L. et al. [23], 2021.	NA	All the equipment 16.3% 1. Disposable syringes 80% 2. Oxygen supply 70% 3. Suction tips 61% 4. Self-inflating balloon 46% 5. Tourniquet 37% 6. AED 26% 7. Magil forceps 20%	All the essential drugs 29.6% 1. Anti-hypoglycemic 70% 2. Antiallergens 68% 3. Antiplatelet 59% 4. Histamine-blockers 56% 5. Vasodilators 53% 6. Bronchodilators 23%	68% had an emergency care protocol;
Al-Sebaei M.O. [34], 2015.	NA	1. Oxygen supply 52.9% 2. Stethoscope 31.4% 3. Glucometer 25.7% 4. Blood pressure device 22.9% 5. Intravenous cannula 21.4% 6. Intravenous line and IV fluid 17.1% 7. Laryngoscope 11.4% 8. Bag-valve mask 10% 9. AED 10% 10. Large suction tip 8.6	1. Adrenaline 48.6% 2. Aspirin 47.1% 3. Nitroglycerine 41.4% 4. Oral glucose 38.6% 5. Hydrocortisone 34.3% 6. Glucagon 30% 7. Bronchodilator 28.6% 8. Oral antihistaminic 15.7% 9. Ammonia inhalant 10%	20% had a written protocol or flow chart for emergency situations.
Alhamad M. et al. [32], 2015.	74.3% had an emergency kit	NA	NA	NA
Umek N. and Šoštarič M. [26], 2018.	A fully prepared emergency kit 4%	1. Pocket mask 60.6% 2. Self-inflating balloon 58.1% 3. Oxygen mask 54.9% 4. Oxygen supply 49.1% 5. Spacer 43% 6. Oropharyngeal airway 40.1% 7. Blood pressure monitor 40.1% 8. Portable suction device 40.1% 9. AED 15.5%	NA	NA
Kaddah M. et al. [8], 2025.	Emergency kit 38.2%	1. Blood pressure monitor 49.9% 2. Glucose meter 26.4% 3. Oxygen supply 14.6% 4. AED 0%	NA	8.8% had a written emergency plan;
Shaath H. et al. [35], 2023.	Had an emergency kit 80%	NA	1. Oral Glucose 69% 2. Adrenalin 68% 3. Hydrocortisone 59% 4. Atropine 39% 5. Ammonia inhalant 31%	NA
Atherton G.J. et al. [36], 1999.	NA	NA	1. Oxygen 95.4% 2. Adrenaline 93.3% 3. Injectable steroids 86.6% 4. Glycerin trinitrate 80.1% 5. Glucose 77.7% 6. Salbutamol inhaler 70%	NA

NA = Data NOT AVAILABLE.

## Data Availability

No new data were created or analyzed in this study. Data sharing is not applicable to this article.

## Supplementary Materials

The following supporting information can be downloaded at: https://www.mdpi.com/article/10.3390/dj14040190/s1, Table S1: PRISMA 2020 Checklist; Table S2: Scoping review search protocol; Table S3: Selected studies and sample characteristics; Table S4: Self-reported confidence and competence.

## Abbreviations

The following abbreviations are used in this manuscript:

ADA	American Dental Association
UK	United Kingdom
AED	Automated External Defibrillator
CPR	Cardiopulmonary Resuscitation
