# Supplementary material for: Medical Emergencies and Operational Preparedness Among Dentists: A Scoping Review
Source: Dent J (Basel). 2026 Mar 24;14(4):190. doi: 10.3390/dj14040190 (PMC13114719; doi:10.3390/dj14040190)
Supplement: Supplementary file 1 [file dentistry-14-00190-s001.zip › dentistry-4121623-supplementary/dentistry-4121623 - supplementary files/Supplementary Table S2.pdf]

Supplementary Table 2. Scoping review search protocol.

Database	Search	Search string	Number of results
**PubMed**	1	"Dentists"[Mesh] OR "Dental Staff"[Mesh] OR "Dental Offices"[Mesh] OR dentist*[tiab] OR dental practitioner*[tiab] OR dental professional*[tiab]	132,851
	2	"Medical Emergencies"[Mesh] OR "Emergency Treatment"[Mesh] OR medical emergency*[tiab] OR emergency situation*[tiab] OR life-threatening emergency*[tiab]	158,065
	3	"Emergency Preparedness"[Mesh] OR "Clinical Competence"[Mesh] OR "Professional Competence"[Mesh] OR "Education, Dental"[Mesh] OR preparedness[tiab] OR readiness[tiab] OR knowledge[tiab] OR training[tiab] OR confidence[tiab] OR competence[tiab]	2,640,364
	4	S1 AND S2	717
	5	S1 AND S3	112,623
	6	S1 AND S2 AND S3	287
**Chochrane Library**	1	(Dentists):ti,ab,kw OR ("dental staff"):ti,ab,kw OR (Dental Offices):ti,ab,kw OR (dental practitioner):ti,ab,kw OR (dental professional):ti,ab,kw (Word variations have been searched)	5232
	2	(Medical Emergencies):ti,ab,kw OR (Emergency Treatment):ti,ab,kw OR (emergency situation):ti,ab,kw OR (life-threatening emergency):ti,ab,kw	18,377
	3	(Emergency Preparedness):ti,ab,kw OR (Clinical Competence):ti,ab,kw OR (Professional Competence):ti,ab,kw OR (Education, Dental):ti,ab,kw	12,166
	4	S1 AND S2	59
	5	S1 AND S3	703
	6	S1 AND S2 AND S3	8
Google Scholar	1	“dentist” OR dentists OR “dental practitioner” OR “dental professionals”	1,550,000
	2	“medical emergency” OR “medical emergencies” OR “emergency situation”	18,400

	3	“preparedness” OR “readiness” OR “knowledge” OR “training” OR “competence” OR “confidence”	8,150,000
	4	S1 AND S2	16,700
	5	S1 AND S3	1.800.000
	6	S1 AND S2 AND S3	15.800
