# Supplementary material for: Medical Emergencies and Operational Preparedness Among Dentists: A Scoping Review
Source: Dent J (Basel). 2026 Mar 24;14(4):190. doi: 10.3390/dj14040190 (PMC13114719; doi:10.3390/dj14040190)
Supplement: Supplementary file 1 [file dentistry-14-00190-s001.zip › dentistry-4121623-supplementary/dentistry-4121623 - supplementary files/Supplementary Table S3.pdf]

Supplementary Table 3. Selected studies and sample characteristics.

Author [ref.], Year.	Study design, Sampling.	Sampled population (SP) Sample size (N) Response rate (RR %)	Sample characteristics	Training
Kaddah M. et al. [8], 2025.	Regional cross-sectional, randomised sampling from the regional database.	SP = 1,821<br>N = 1,034<br>RR = 56.8%	**Profession**<br>General dentists 70.7%<br>Dental specialists 23.4%<br>Dental residents 5.9%<br><br>**Practice**<br>Private office 70.6%<br>Public-owned office 11.7%<br>Both 17.7%<br><br>**Workplace**<br>Rural 55.8%<br>Urban 44.2%	32.1% had formal training;<br>50.1% din not receive any emergency training;
Subhadra H.N. et al. [7], 2025.	Regional cross-sectional, randomised sampling.	SP = 884<br>N = 609<br>RR = 68.9%	NA	78.8% had undergraduate training;<br>82.3% had BLS training;<br>13.1% trained at 2 year interval;
Choufani et al. [9], 2025.	National cross-sectional, sampled the entire database (online-based questionnaire).	SP = 7000<br>N = 442<br>RR = 6.31%	**Profession**<br>General practitioners 40%<br>Residents 5.4%<br>Other specialities 54.3%<br><br>**Practice**<br>Independent practice 71.9%<br>Shared practice 43.6%<br>A university 20.4%	71.3% received undergraduate training;<br>39.6% received postgraduate training;<br>28.5% were never instructed;

			Hospital practice 4.8%	
Špiljak B. et al. [37], 2024.	National cross-sectional study, sampled the entire dentist population.	SP = NA<br>N = 319<br>RR = NA	**Profession**<br>General practitioners 77.7%<br>Oral Surgery 5.3%<br>Dental Prosthetics 4.1%<br>Periodontology 0.9%<br>Endodontics 2.8%<br>Other 9.2%<br>**Practice**<br>Private clinic 59.2%<br>Dental Polyclinic 4.5%<br>School of Dental Medicine 4.5%<br>Hospital 1%	47.6% attended any training
Gupta S. et al. [38], 2023.	National cross-sectional study,<br>sampling strategy unknown.	SP = NA<br>N = 500<br>RR = NA	**Profession**<br>General practitioners	44.4% atended any training.
Varoni et al. [1], 2023.	National cross-sectional, sampled the entire dentist population.	SP = 62,441<br>N = 6818<br>RR = 10.9%	**Practice**<br>Private Practice 88.4%<br>Public Practice 11.6%	44.1% received undergraduate training;<br>72.9% received postgraduate training;
Sin M. et al. [31], 2023.	National cross-sectional, sampling strategy unknown.	SP = NA<br>N = 400<br>RR = NA	**Profession**<br>Dentists 55.8%<br>Dental Hygienists 38.8%<br>Dental specialists 5.5%<br>**Practice**<br>Private practice 54.5%<br>Primary dental care 86.8%	89.2% undertake medical emergency training each year;<br>62% received BLS training within the last year;<br>50.5% theoretical medical emergency training;<br>32% simulation mannequin training;<br>50.5% medical emergency roleplay;
Shaath H. et al. [35], 2023.	National cross-sectional, sampling strategy unknown.	SP = 100<br>N = 97<br>RR = 97.0%	**Profession**<br>General practitioners 66%<br>Other speciality 34%	NA

Al Ghanam et al. [21], 2022.	National cross-sectional, sampling strategy unknown.	SP = NA<br>N = 180<br>RR = NA	**Profession**<br>General dentistry 51.1%<br>Surgeons 20.9%<br>Pedodontists 15.4%<br>Orthodontists 14.3%<br>Conservative dentists 13.2%<br><br>**Practice**<br>Independent practice 16.1%<br>Shared practice 17.2%<br>Hospital/clinics 53.9%	27.7% received undergraduate training;<br>61.1% received postgraduate training;<br>27.1% were trained within the previous year;
Jaber L. et al. [23], 2021.	National cross-sectional, random sampling.	SP = 335<br>N = 270<br>RR = 80%	NA	52.6% had BLS certification;
Jing Q., et al. [18], 2020,	National cross-sectional, sampled course attendants.	SP = 2408<br>N = 2042<br>RR = 84.3%	**Profession**<br>Dentists	84% had never received training other than BLS;
Smereka et al. [28], 2019.	National cross-sectional, sampling strategy unknown.	SP = 700<br>N = 419<br>RR = 60.3%	**Profession**<br>General dentists<br><br>**Practice**<br>Independent practice 72.55%<br>Clinics 34.13%<br>Hospital 5.73%	8.35% never received training after graduation;<br>24.82% received training within the last year;
Umek N. and Šoštarič M. [26], 2018.	National cross-sectional, sampled the entire population.	SP = 1503<br>N = 289<br>RR = 19.2%	**Profession**<br>General dentistry 90%<br>Other specialities 10%<br><br>**Practice**<br>State owned 50.5%<br>Independent practice 48.1%<br>Part of a dispensary 50.9%<br>Hospital 1%	94.3% received undergraduate training;<br>85.1% received postgraduate training;<br>74.3% were trained to use an AED;<br>14.9% never received BLS training;

Kumarswami S. et al. [33], 2017.	Regional cross-sectional, random sampling.	SP = 250<br>N = 250<br>RR = 100%	NA	7.6% attended any training.
Čuković-Bagić I. et al. [19], 2017.	National cross-sectional, sampling strategy unknown.	SP = 525<br>N = 498<br>RR = 94.9%	**Profession**<br>Dentists	71.3% received adult BLS training during undergraduate training;<br>38.1% received training after graduation;
Geguzis et al. [30], 2016.	National cross-sectional, random sampling.	SP = 510<br>N = 412<br>RR = 80.8%	**Profession**<br>General dentistry 57.8%<br>Prosthodontist 12.9%<br>Orthodontist 10.4%<br>Oral Surgeons 6.3%<br>Paediatric Dentists 5.1%<br>Endodontist 3.9%<br>Periodontology 3.6%<br><br>**Practice**<br>Independent practice 53.6%<br>State policlinic 18%<br>Both 28.4%<br><br>**Workplace**<br>Rural 17%<br>Urban 83%	93.9% received some form of training;
Joshi S. et al. [22], 2015.	National cross-sectional, sampled participants of a conference.	SP = 200<br>N = 124<br>RR = 62%	**NA**	61.3% received undergraduate training in BLS;<br>15.3% received postgraduate training;
Müller et al. [27], 2015.	Regional cross-sectional, sampled the entire population.	SP = 2998<br>N = 620<br>RR = 21%	**Practice**<br>Individual practice 71%<br>Shared practice 17%<br>Co-operative 12%<br>Hospital/policlinics 17%<br><br>**Workplac**e	92% were trained;<br>23% received only one training;<br>68% were exposed to more than one training;<br>18% were never trained;

			Rural 23%<br>Urban 77%	
Al-Sebaei M.O. [34], 2015.	Regional cross-sectional, random sampling.	SP = NA<br>N = 70<br>RR = NA	NA	NA
Alhamad M., et al. [32], 2015.	Regional cross-sectional, random sampling.	SP = 198<br>N = 145<br>RR = 73.2%	**Practice**<br>Independent practice 56%<br>State-owned practice 44%	NA
Laurent et al. [20], 2014.	National cross-sectional, sampled the entire population.	SP = 25535<br>N = 1344<br>RR = 6.5%	**Profession**<br>General dentists 95.5%<br>Orthodontists 2.8%<br>Oral surgeons 3%<br><br>**Practice**<br>Individual practice 46.1%<br>Shared practice 41.1%<br>Hospital 8.9%<br><br>**Workplace**<br>Urban 83.3%<br>Rural 11.2%<br>Both 5.6%	57.1% received training during their university studies;<br>64.3% s postgraduate training;<br>8.7% were never instructed;<br>14.3% were trained within the last 12 months;
Marks L. A. M. et al. [17], 2013.	National cross-sectional, sampled conference participants.	SP = NA<br>N = 548<br>RR = NA	**Profession**<br>Dentists<br><br>**Practice**<br>Private practice 61.1%<br><br>**Workplace**<br>Rural 36.1%<br>Urban 63.5%	38.5% undergraduate BLS training;<br>38.5% had postgraduate training;<br>49.4% never participated in any BLS training;

Arsati et al. [25], 2010.	National cross-sectional study, sampled conference participants.	SP = NA<br>N = 498<br>RR = NA	**Practice**<br>Private practices 50.8%<br>State-owned practices 12%<br>Both 33.5%	59.6% received some form of training, of which 70% were trained only once;<br>29.9% received undergraduate training;<br>21.2% received postgraduate training;
Broadbent J.M. and Thomson E.M. [29], 2001.	National cross-sectional, random sampling.	SP = 450<br>N = 199<br>RR = 63.4%	NA	81.8% undergraduate training;<br>77.8% postgraduate first aid training;<br>57.6% postgraduate medical emergency training;<br>35.4% other emergency postgraduate courses;
Atherton G.J. et al.* [24, 36, 41], 1999.	National cross-sectional, sampling strategy unknown.	SP = 1500<br>N = 1110<br>RR = 74%	NA	75% undergraduate training in medical emergencies;<br>95% received postgraduate training;<br>14.8% reported drills within their practice;
Chapman P.J. et al. [16], 1997.	National cross-sectional, random sampling.	SP = 1250<br>N = 811<br>RR = 65%	NA	64% received postgraduate CPR training;<br>20% had CPR training within the last year;

NA = Data NOT AVAILABLE;
* Study in reference 41 only reflects sample demographic characteristics on training exposure and was not included in the sampled data on emergency prevalence or operational preparedness, but is presented here for contextualisation.
