# Supplementary material for: Medical Emergencies and Operational Preparedness Among Dentists: A Scoping Review
Source: Dent J (Basel). 2026 Mar 24;14(4):190. doi: 10.3390/dj14040190 (PMC13114719; doi:10.3390/dj14040190)
Supplement: Supplementary file 1 [file dentistry-14-00190-s001.zip › dentistry-4121623-supplementary/dentistry-4121623 - supplementary files/Supplementary Table S4.pdf]

Supplementary Table 4. Self-reported confidence and competence.

Author [ref.]	Diagnose medical emergencies	Treat medical emergencies	Perform CPR / other medical procedures
Chapman P.J. et al. [16]	NA	NA	55% declared competence in CPR;
Marks L. A. M. et al. [17]	Most felt competent to diagnose;	Most felt competent to treat minor medical emergencies;<br>Most felt incompetent to treat heart attack, cardiac arrest, and anaphylaxis;	NA
Arsati et al. [25]	41% were confident in diagnosing an emergency event;	More than half declared they were incapable of initial management for emergencies such as Angina, Asthma, Hyperventilation crises, cardiac arrest, myocardial infarction, cerebrovascular accidents, hypertension crises, and anaphylaxis;	More than half declared a lack of competence in performing CPR and administering intravenous medication;
Čuković-Bagić I. et al. [19]	20.5% were unable to diagnose the emergency situation;	57% do not feel confident in managing cardiac arrest;<br>Up to and more than half of the participants do not feel confident in managing most medical emergencies;	NA
Špiljak B. et al. [37]	NA	25.9% were prepared or extremely prepared for any medical emergency;<br>18.2% were prepared for cardiac arrest;<br>More than half declared being prepared for vasovagal syncope (76%), hypoglycaemia (63.5%), hyperventilation crises (57.1%), and epileptic seizures (53.5%);<br>Less than half reported being prepared for toxin reaction to anaesthetics (22.2%), Vasoconstrictor reactions (27.9%), anaphylaxis (28.1%), angina (31.2%), myocardial infarction (23.4%), asthma attack (35.1%), and foreign body inhalation (28.2%);	More than half were prepared to use bronchodilators, aspirin, nitroglycerine, and diazepam;<br>Most displayed low preparedness for the use of emergency medical devices;
Laurent et al. [20]	NA	Most participants were confident in treating minor emergencies (88.7%);<br>More than half (55.1%) were confident they could treat a serious emergency;	NA

		Only 36.3% felt confident to handle a cardiac arrest.	
Müller et al. [27]	NA	NA	Perform bag/mask ventilation 57% ;<br>Perform BLS 49%;<br>Perform airway management 16%;<br>perform ALS 9%;<br>Defibrillate 3%;
Subhadra H.N. et al. [7]	NA	NA	53.5% felt confident to administer IM and IV drugs;<br>47.3% felt confident to perform chest compressions;<br>46.5% felt confident in administering mouth-to-mouth resuscitation;
Gupta S. et al. [38]	NA	69.9% were confident in dealing with an emergency situation;	77.4% declared competence in performing CPR;
Kumarswami S. et al. [33]	NA	94% felt confident to handle an emergency situation;	34% felt confident to administer intramuscular medication;<br>6.6% felt confident administering intravenous medication;
Varoni et al. [1]	NA	Most participants lack confidence in treating more severe emergent conditions such as myocardial infarction, transient ischemic attack, and stroke.	NA
Al Ghanam et al. [21]	NA	Most participants were confident in treating minor emergencies (66.1%);<br>Most participants were not confident in resolving major emergencies (65%);<br>Only 18.8% felt confident to handle cardiac arrest;	NA
Choufani et al. [9]	NA	Respondents reported low preparedness to respond to severe medical emergencies.	NA
Geguzis et al. [30]	NA	NA	41.5% can use emergency equipment;<br>31.8% were not confident they could resuscitate;

Joshi S. et al. [22]	NA	44.4% manage emergency situations at least well;	28.2% were confident that they could use emergency drugs;<br>50.8% were not confident in administering intravascular or intramuscular medication;
Broadbent J.M. and Thomson E.M. [29]	NA	37.9% felt well prepared for medical emergencies;	Most felt competent in the use of medical devices and drugs;
Smereka et al. [28]	NA	Most respondents (>50%) declared competence in managing cardiac arrest, syncope, orthostatic Hypotension, mild allergic reaction, angina, and hypoglycaemia.<br>Less than half declared competence in managing anaphylactic shock, seizure, hypertensive crisis, and asthma.	NA
Jaber L. et al. [23]	50% felt confident in recognizing a medical emergency;	49% were confident they could prevent a medical emergency;<br>48% felt confident to manage a medical emergency;	NA
Al-Sebaei M.O. [34]	NA	The mean score for preparedness to act in an emergency was minimally above average;	NA
Alhamad M., et al. [32]	NA	NA	44.8% felt competent to perform CPR.
Umek N. and Šoštarič M. [26]	Most participants reported strong confidence in diagnosing syncope;<br>Most participants reported low competence in diagnosing stroke, cardiac arrest, and hypertensive crisis;	Most participants reported strong confidence in managing syncope;<br>Most participants reported low competence in managing stroke, cardiac arrest, and hypertensive crisis;<br>Participants felt more confident in managing than diagnosing medical emergencies;	49.4% were not confident in performing CPR;<br>60.4% were not confident in establishing venous access;
Kaddah M. et al. [8]	NA	The majority reported moderate confidence (52.9%), while 11.7 described excellent confidence;<br>43.9% considered themselves to possess good or excellent knowledge in emergency care;	NA

Shaath H. et al. [35]	NA	51% affirmed they could manage syncope, anaphylactic shock, and foreign body inhalation;	75% felt competent to administer intramuscular injection;<br>47% felt competent to administer intravenous medication;
Sin M. et al. [31]	NA	Participants reported elevated confidence in managing most procedures, except for adrenal crisis;	Participants' confidence level in performing emergency procedures was elevated for most procedures, except for inserting an oral airway.
Atherton G.J. et al. [41]	NA	Participants who were not trained as undergraduates were likely to feel not prepared for emergencies;<br>20% felt unprepared for medical emergencies;	NA

NA = Data NOT AVAILABLE;
